# A comparison of clinical guidelines, treatment characteristics and outcomes in small cell lung cancer between East Asia and Europe/North America

**DOI:** 10.3389/fonc.2025.1646608

**Published:** 2025-10-27

**Authors:** Chi-Lu Chiang, Ross Andrew Soo, Tony Mok, Pei Jye Voon, Lucksamon Thamlikitkul, Ying Cheng, Hidehito Horinouchi, Byong Chul Cho, Krista Lin Xu, Myung-Ju Ahn

**Affiliations:** ^1^ Department of Chest Medicine, Taipei Veterans General Hospital, Taipei, Taiwan; ^2^ School of Medicine, National Yang Ming Chiao Tung University, Taipei, Taiwan; ^3^ Department of Haematology-Oncology, National University Hospital, Singapore, Singapore; ^4^ Department of Clinical Oncology, Faculty of Medicine, State Key Laboratory in Oncology in South China, The Chinese University of Hong Kong, Hong Kong, Hong Kong SAR, China; ^5^ Department of Radiotherapy and Oncology, Sarawak General Hospital, Kuching, Sarawak, Malaysia; ^6^ Division of Medical Oncology, Department of Medicine, Faculty of Medicine Siriraj Hospital, Mahidol University, Bangkok, Thailand; ^7^ Jilin Cancer Hospital, Changchun, China; ^8^ Department of Thoracic Oncology, National Cancer Center Hospital, Tokyo, Japan; ^9^ Yonsei Cancer Center, Yonsei University College of Medicine, Seoul, Republic of Korea; ^10^ Amgen, Inc., Singapore, Singapore; ^11^ Division of Hematology-Oncology, Department of Medicine, Samsung Medical Center, Sungkyunkwan University School of Medicine, Seoul, Republic of Korea

**Keywords:** Asian countries, East Asia, diagnosis, small cell lung cancer, survival outcomes, treatment guidelines, treatment patterns

## Abstract

Small cell lung cancer (SCLC) is an aggressive neuroendocrine carcinoma with a poor prognosis and accounts for approximately 11% of all lung cancers. Owing to the complex and aggressive nature of the disease, clinical management of SCLC is challenging. Many SCLC regional guidelines, including those from East Asia, have been developed in light of potential regional variations in socioeconomic conditions and healthcare infrastructure. However, less is known about the potential implications of the inherent population/regional differences in clinical management and the emerging treatment landscape in SCLC. Here, we review variations in the real-world patient characteristics and in diagnosis and treatment guidelines in SCLC between East Asia and Europe/North America. We also consider similarities and differences in real-world treatment patterns, as well as clinical outcomes between regions, to explore the need to adapt clinical management in SCLC.

## Introduction

Small cell lung cancer (SCLC) is an aggressive neuroendocrine (NE) carcinoma, characterized by rapid proliferation, a predisposition for early metastasis, and poor prognosis (1). SCLC accounts for approximately 11% of all lung cancers, and the survival rate is poor (2–4). Early diagnosis is crucial, yet challenging owing to the aggressive nature of the disease, which limits options for curative treatment (1, 5). SCLC is linked with tobacco smoking, and its prevalence often follows the trend in smoking prevalence, with a lag period of approximately 30 years (5).

SCLC carcinogenesis involves multiple pathways, including those disrupting normal DNA repair mechanisms, leading to genomic instability (6). Patients with a history of smoking are likely to have a high tumor mutational burden (TMB) in SCLC (7, 8). Genomic analyses have shown that the most frequent mutations and chromosomal aberrations in patients with SCLC involve inactivation of the tumor protein 53 (TP53) and/or loss of retinoblastoma 1 (RB1) genes (9, 10). Deregulation of the Notch pathway has also been shown to contribute to the clinical behavior of SCLC, including drug resistance and relapse (11, 12). Notch-1 receptor-mediated processes such as NE differentiation, proliferation, cell adhesion, and epithelial to mesenchymal transition play a key role in SCLC development and tumorigenesis (11).

Although SCLC is considered a single disease entity, there are biologically distinct subtypes due to complex pathophysiology and tumor heterogeneity. Complex associations between NE expression and transcription factors warrant further investigation owing to potential subtype-specific therapeutic vulnerabilities (13, 14). A key area that has been poorly understood in SCLC is the influence of ethnic or regional variations in patient characteristics, in the diagnosis and treatment of SCLC, including real-world treatment patterns, and in clinical outcomes. Owing to potential regional variations in socioeconomic conditions and healthcare infrastructure, different regional guidelines for the treatment of SCLC have been developed (15). Although these guidelines are primarily based on the American Joint Committee on Cancer (AJCC) Tumor, Node, Metastasis (TNM) classification (stages 0–IV), most have adopted a pragmatic approach of combining the TNM staging system and the previous Veterans Administration (VA) two-stage classification scheme (i.e., limited-stage [LS] disease and extensive-stage [ES] disease) (15).

Unlike in non–small cell lung cancer (NSCLC), there are no pan-Asian guidelines developed for SCLC diagnosis and management (16). Additionally, publications comparing SCLC guidelines from across the globe are limited. In this narrative review, we focused on comparing East Asia with Europe and/or North America (Europe/North America). We provide a comparison of real-world patient characteristics and diagnosis and treatment guidelines between East Asia and Europe/North America. We further explore similarities and differences in real-world treatment patterns, as well as clinical outcomes in SCLC, to shed some light on the potential implications of the inherent population/regional differences in clinical management and the emerging treatment landscape.

## Real-world patient characteristics in SCLC: East Asia versus Europe/North America

Smoking is associated with SCLC, with most patients being former or current smokers (6, 17). Besides smoking history, patient characteristics such as sex and age are potential risk factors associated with SCLC (**Table 1**). Studies from East Asia indicated continued increased prevalence of SCLC in men compared with women (18–20). In contrast, recent reports from Europe and the United States (US) suggest a shift from the initial male predominance of SCLC to an equal prevalence in men and women (12, 15, 21). There is also an increased prevalence of SCLC in elderly populations (>70 years of age) compared with younger age groups, a trend similar in East Asia and Europe/North America (18, 22).

**Table 1:** SCLC patient characteristics in East Asia versus Europe/North America.

Characteristics	East Asia		Europe/North America	
Current smokers/smoking history				
%	**Range^a^:**	48.3–97.6 (32–46)	Range: 38.7–98.2 (17, 24–31)	
	China:	55.4–77.2 (32–36, 45)		
	Japan:	48.3–97.6 (37–39, 46)		
	Korea:	71.9–85.2 (40–42)		
	Taiwan:	78.9 (43)		
	Thailand:	90.7 (44)		
Age, % (n)	Korea:	<65 years: 48.7 (166) (49)	<80 years: 91.8 (5,078)^a^ (28)	
		≥65 years: 51.3 (175) (49)	≥80 years: 8.2 (452)^a^ (28)	
		≥65 years: 69.7 (620)^a^ (41)		
	Taiwan:	<70 years: 55.2 (2,758)^a^ (43)		
		70 years: 44.8 (2,242)^a^ (43)		
Sex, % (n)	Korea:	M: 95.0 (324); F: 5.0 (17) (49)	M: 57.0 (1,249); F: 15.0 (331) (17)	
		M: 17.4 (270)^a^; F: 23.9 (22)^a^ (42)	M: 47.6 (2,632)^a^; F: 52.4 (2,898)^a^ (28)	
		F: 7.0 (62)^a^ (41)		
	Taiwan:	M: 93.6 (4,678)^a^; F: 6.4 (322)^a^ (43)		
ECOG PS, % (n)	Korea:	0–1: 93.5 (319) (49)	0–1: 31.0 (1,713) (28)	
		≥2: 6.5 (22) (49)	≥2: 12.5 (692) (28)	
		≥2: 16.8 (113)^a^ (41)		
	Taiwan:	0–1: 52.8 (2,641)^a^ (43)		
		≥2: 25.2 (1,261)^a^ (43)		
Stage, % (n)	Korea:	LS: 45.5 (155) (49)	LS: 31.8 (1,761)^a^ (28)	
		ES: 54.5 (186) (49)	ES: 62.2 (3,440)^a^ (28)	
		LS: 39.2 (112)^a^ (42)	LS: 54.3 (75) (24)	
		ES: 60.8 (174)^a^ (42)	ES: 50.6 (43) (24)	
		LS: 36.6 (325)^a^ (41)		
		ES: 57.8 (514)^a^ (41)		
	Taiwan:	I–III: 28.2 (1,410)^a^ (43)		
		IV: 71.8 (3,590)^a^ (43)		
Ex-smokers^b^				
%	**Range**:	23.6–48.8 (37, 40)	**Range**: 23.6–48.6 (17, 24, 25, 27, 29, 30)	
	Japan:	48.8 (37)		
	Korea:	23.6 (40)		
Sex, % (n)	–		M: 21.0 (2,201) (17)	
			F: 3.0 (2,201) (17)	
Stage, % (n)	–		LS: 42.8 (59) (24)	
			ES: 47.1 (40) (24)	
Never-smokers				
%	**Range^a^:**	1.9–44.6 (32, 34–46, 49)	**Range:** 1.5–4.5 (17, 24–30, 48, 50, 51)	
	China:	22.8–44.6 (32, 34–36, 45)		
	Japan:	2.4–10.8 (37–39, 46)		
	Korea:	3.4–16.9 (40–42, 49)		
	Taiwan:	13.5 (43)		
	Thailand:	1.9 (44)		
Age, % (n)	Korea:	<65 years: 40.0 (20) (49)	<80 years: 83.0 (83) (28)	
		≥65 years: 60.0 (30) (49)	≥80 years: 17.0 (17) (28)	
		≥65 years: 81.8 (126) (41)		
	Taiwan:	<70 years: 42.7 (366) (43)		
		≥70 years: 57.3 (492) (43)		
Sex, % (n)	Korea:	M: 20.0 (10); F: 80.0 (40) (49)	M: 1.0 (22); F: 2.3 (50) (17)	
		M: 9.5 (28); F: 4.7 (30) (42)	M: 34.0 (34); F: 66.0 (66) (28)	
		F: 50.6 (78) (41)	M: 12.5 (4); F: 87.5 (28) (120)	
	Taiwan:	M: 68.1 (584); F: 31.9 (274) (43)		
ECOG PS, % (n)	Korea:	0–1: 86.0 (43) (49)	0–1: 30.0 (30) (28)	
		≥2: 14.0 (7) (49)	≥2: 13.0 (13) (28)	
		≥2: 21.6 (24) (41)	0–1: 56.3 (18) (120)	
	Taiwan:	0–1: 41.3 (354) (43)	≥2: 28.1 (9) (120)	
		≥2: 33.1 (284) (43)		
Stage, % (n)	Korea:	LS: 52.0 (26) (49)	LS: 20.0 (20) (28)	
		ES: 48.0 (24) (49)	ES: 70.0 (70) (28)	
		LS: 24.1 (14) (42)	LS: 53.1 (17) (120)	
		ES: 75.9 (44) (42)	ES: 46.9 (15) (120)	
		LS: 30.5 (47) (41)	LS: 2.9 (4) (24)	
		ES: 66.2 (102) (41)	ES: 2.4 (2) (24)	
	Taiwan:	I–III: 23.1 (198) (43)		
		IV: 76.9 (660) (43)		

^a^Current or ex-smokers not defined; ^b^Data on age and ECOG PS not available.

ECOG PS, Eastern Cooperative Oncology Group performance status; ES, extensive-stage; F, female; LS, limited-stage; M, male; SCLC, small cell lung cancer.

Among the global smoking population, the prevalence of smoking in men was highest in East and South-East Asia and East Europe, and the highest prevalence in women was noted in European countries (23). Given the link between smoking and SCLC, the prevalence of SCLC seems to mirror the prevalence of smoking (5). The proportion of smokers with SCLC ranged from 48.3 to 97.6% (year of publication, range: 2015–2023) in East Asia versus 38.7–98.2% in Europe/North America (year of publication, range: 2012–2023) (17, 24–46) (**Table 1**). Although the relative incidence of SCLC has declined over the past few decades, reflecting a decrease in smoking prevalence, studies suggest that the risk of developing SCLC in young smoking populations is on the rise (6, 12, 17). It should be noted that there is a lag time of approximately 30 years between smoking and occurrence of SCLC; hence, any variations in the prevalence of SCLC are likely attributable to the differences in smoking over time (5, 23, 47).

Although SCLC is linked to smoking, SCLC can occur in never-smokers (43, 48, 49). There are regional- and sex-based differences in the prevalence of SCLC in never-smokers. The East Asian population has a higher incidence of SCLC among never-smokers compared with the European/North American population. Based on available data, the proportion of never-smokers with SCLC ranged from 1.9 to 44.6% in East Asia (year of publication, range: 2015–2023) versus 1.5–4.5% in Europe/North America (year of publication, range: 2009–2023) (17, 24–30, 32, 34–46, 48, 50, 51) (**Table 1**). The higher incidence of SCLC among never-smokers in East Asia versus Europe/North America may be attributed to ethnic differences, second-hand smoking, and increased exposure to occupational and environmental carcinogens in East Asia (52). Regardless of these regional differences, women account for a high proportion of never-smoking patients with SCLC in both East Asia and Europe/North America (17, 32, 49, 52). Although data indicate the presence of distinct molecular profiles in never-smokers with SCLC compared with those with a smoking history, less is known about any regional differences in the prevalence of molecular subtypes of SCLC in East Asia versus in Europe/North America (28). Although the Achaete-scute homolog 1 NE subtype seems to be the most prevalent subtype of SCLC, on the basis of studies from East Asia and Europe/North America, further comparative analyses are needed to reveal the existence of any distinct mutational signatures in these regional populations (9, 53–59). A study by Lin et al. indicated potential disparities in mutational signatures in East Asian patients with SCLC versus White patients (60). The observation that the East Asian study population had high mutation counts of DNA-damage response signaling pathways and TMB compared with the White study population (P<0.05) may have important therapeutic implications (60).

## Diagnosis and staging of SCLC in East Asia versus Europe/North America

SCLC is typically diagnosed when patients present with symptoms indicative of locally advanced or metastatic-stage disease (61, 62). There is no effective screening test available to detect early-stage SCLC (61, 62). Low-dose computed tomography (CT) screening has been shown to reduce lung cancer mortality in asymptomatic high-risk patients (63). However, this screening test is not an effective approach for SCLC detection, because of the symptomatic development of the disease between annual CT scans, owing to its aggressive nature (21, 62).

Guidelines regarding the diagnosis of SCLC are generally similar across East Asia and Europe/North America, and recommend a combination of imaging and pathological examination (21, 62, 64–66). These guidelines primarily follow the World Health Organization (WHO) classification system for lung tumors, which is based on the characteristic histology on hematoxylin and eosin staining when good-quality histologic samples are available (67). Mitotic cell counting is essential for differential diagnosis, and the WHO suggests immunohistochemistry (IHC) as a supportive tool in SCLC definitive diagnosis (67). Although international guidelines are in general agreement regarding diagnostic approaches, there is limited concordance among pathologists on ideal diagnostic criteria (68).

In Europe, histological examination of a biopsy is recommended for SCLC diagnosis by the European Society for Medical Oncology (ESMO) (21). Although the ESMO Guidelines note the use of NE markers such as synaptophysin, chromogranin A, neural cell adhesion molecule (NCAM/CD56), and the nuclear protein Ki-67, a recommendation for the use of specific markers is lacking (21, 69). There is no established role for the use of molecular testing in Europe, and programmed cell death ligand 1 (PD-L1) and TMB testing are not recommended in routine clinical practice (21).

In North America, diagnosis can be based on biopsy or cytology of a primary or metastatic site (62, 70). Based on the guidelines developed by the National Comprehensive Cancer Network^®^ (NCCN^®^), if a sample is limited, IHC is recommended for SCLC diagnosis and for distinguishing from NSCLC or other NE tumors (62). Markers for IHC, such as insulinoma-associated protein 1 (INSM1), chromogranin A, NCAM/CD56, and synaptophysin, are suggested, although these alone are not recommended for SCLC diagnosis (62). The NCCN Clinical Practice Guidelines in Oncology (NCCN Guidelines^®^) suggest molecular profiling via blood, tissue or both in rare cases of SCLC, particularly for patients with ES-SCLC or relapsed SCLC who are never-smokers, light-smokers, or who have a remote smoking history (62). Similarly, the Canadian consensus report on SCLC management suggests molecular testing for driver mutations when combined SCLC (defined as a combination of SCLC and non–small cell carcinoma of any histological type) is suspected or in nonsmokers with a new diagnosis of SCLC (5, 70).

Most East Asian countries generally follow the ESMO and/or NCCN Guidelines^®^ for SCLC diagnosis. In Taiwan and Thailand, for example, SCLC diagnosis is based predominantly on NCCN Guidelines, whereas in China and Korea, recommendations around diagnosis are broadly based on the NCCN and ESMO Guidelines. In China, histopathology- and cytology-based diagnosis is recommended to detect the presence and type of tumor (66). Japan follows a similar diagnostic pathway to other countries, which involves imaging and pathological examination (64). On the basis of the Japan Lung Cancer Society Guidelines, pathological diagnosis using NE markers such as chromogranin A, synaptophysin, and NCAM/CD56 are currently used to distinguish SCLC from other lung NE tumors in Japan (64).

Once SCLC is diagnosed, staging of the disease is an important factor when considering the appropriate treatment. The guidelines reported in this article have adopted a combined approach for staging SCLC using both the AJCC TNM staging system and the VA two-stage classification scheme (21, 62, 64–66, 71). However, there seems to be a lack of consensus for the classification of LS- and ES-SCLC (15). Descriptions used for defining LS and ES disease either lack clarity or are inconsistent among these guidelines. Of note, in their definition of LS-SCLC, NCCN Guidelines exclude T3–T4 owing to multiple lung nodules that either are too extensive or have tumor/nodal volume that is too large to use a tolerable radiation plan, unlike ESMO, Chinese, and Taiwanese guidelines (21, 62, 65, 66). The Japanese guidelines have primarily adopted the VA two-stage, classification-based definitions of “localized SCLC and extensive SCLC,” especially when considering treatment choice (64). The rationale for this is based on applicability of this staging criteria in treatment choice and the widespread usage of these terms in clinical trials (64).

## Current treatment of SCLC in East Asia versus Europe/North America

### Comparison of treatment guidance across regions and key differences in recommended therapies in LS- and ES-SCLC

**Figure 1:**
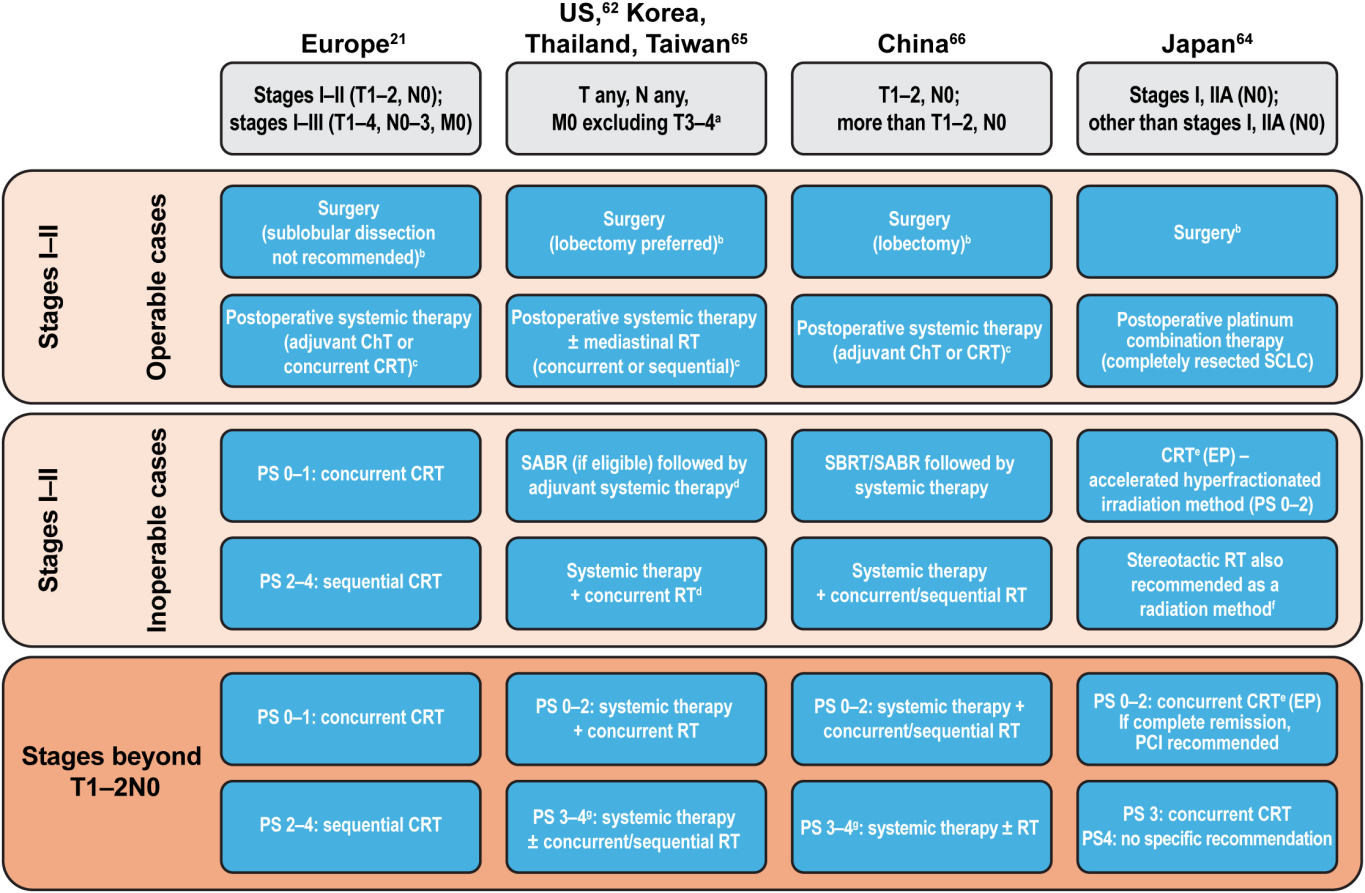
Summary of treatment guidance in LS-SCLC across East Asia and Europe/North America. ^a^Due to multiple lung nodules that are either too extensive or have tumor/nodal volume that is too large to use a tolerable radiation plan; ^b^Pathological mediastinal staging recommended; ^c^Depending on postoperative clinical stage; ^d^SABR not included in management consensus for Taiwan; ^e^Recommended method; if ineligible for EP, then sequential therapy with EC followed by RT; ^f^In stages I–IIA (TNM 9th edition) without lymph node metastases (weak recommendation); ^g^Due to SCLC. ChT, chemotherapy; CRT, chemoradiotherapy; EC, etoposide + carboplatin; ECOG, Eastern Cooperative Oncology Group; EP, etoposide + cisplatin; (LS-)SCLC, (limited-stage) small cell lung cancer; PS, ECOG performance status; RT, radiotherapy; SABR, stereotactic ablative radiotherapy; SBRT, stereotactic body radiation therapy; TNM, Tumor, Node, Metastasis; US, United States.

A comparison of treatment recommendations in LS- and ES-SCLC highlights some differences between regional guidelines. As with SCLC diagnosis guidance, Korea, Taiwan, and Thailand follow the NCCN Guidelines for SCLC treatment, whereas China follows the Chinese Society of Clinical Oncology Guidelines (66). As noted earlier, differences between these guidelines largely lie within how each country or region defines a clinical stage eligible for the recommended treatment algorithm (21, 62, 64–66). A summary of treatment guidance across East Asia and Europe/North America in LS-SCLC is provided in **Figure 1**. Treatment guidelines on ES-SCLC largely align across regions.

Globally, there is a general consensus in the overall approach to treating SCLC. Chemoradiotherapy is recommended for LS-SCLC treatment, especially for patients who cannot undergo surgery, and systemic therapy using a combination of chemotherapy and immunotherapy is generally recommended for the treatment of ES-SCLC (21, 62, 64–66). However, there are some differences in the choice of drugs used across East Asia and Europe/North America in LS- and ES-SCLC (**Table 2**). It must be also noted that any recent approvals of indications in SCLC might not have been updated in the guidelines during the development of this report.

**Table 2:** Comparison of first-line and second-line guideline-recommended systemic treatments for LS- and ES-SCLC: East Asia versus Europe/North America.

	China (66)	Japan (64)	Taiwan (65)	Europe (21)	US/Korea/Thailand (62)
1L					
LS-SCLC					
	Cisplatin + etoposide; etoposide + carboplatin	Cisplatin + etoposide; cisplatin + irinotecan	Cisplatin + etoposide^a^; carboplatin + etoposide; Adjuvant therapy with durvalumab after concurrent or sequential chemotherapy for 24 months^a^	Cisplatin + etoposide^a^; carboplatin + etoposide^b^	Cisplatin + etoposide^a^; consolidation therapy: durvalumab^a^; carboplatin + etoposide^b^
ES-SCLC					
	**ECOG PS 0–2** **ECOG PS 3–4 (SCLC related):**	**ECOG PS 0–1:**	**ECOG PS 0–1:**	**ECOG PS 0–1 (if no contraindications for IO):**	Carboplatin + etoposide + atezolizumab followed by maintenance atezolizumab^a^ Carboplatin + etoposide + atezolizumab followed by maintenance lurbinectedin + atezolizumab^a^
	Carboplatin + etoposide + atezolizumab followed by maintenance atezolizumab	Carboplatin + etoposide + atezolizumab followed by maintenance atezolizumab^a^	Carboplatin + etoposide + atezolizumab followed by maintenance atezolizumab^a^	Carboplatin + etoposide + atezolizumab followed by maintenance atezolizumab^a^	Carboplatin/cisplatin + etoposide + durvalumab followed by maintenance durvalumab^a^
	Carboplatin/cisplatin + etoposide + durvalumab followed by maintenance durvalumab	Carboplatin/cisplatin + etoposide + durvalumab followed by maintenance durvalumab^a^/irinotecan	Carboplatin/cisplatin + etoposide + durvalumab followed by maintenance durvalumab^a^	Carboplatin/cisplatin + etoposide + durvalumab and maintenance durvalumab^a^	Carboplatin/cisplatin + etoposide; carboplatin/cisplatin + irinotecan
	Carboplatin + etoposide + serplulimab followed by maintenance serplulimab	**ECOG PS 2:** Platinum + etoposide or irinotecan	Carboplatin/cisplatin + etoposide	**ECOG PS 0–1 (contraindications for IO):**	
	Carboplatin + etoposide + adebrelimab followed by maintenance adebrelimab	**ECOG PS 0–2, ≤70 years of age:**		Carboplatin + etoposide; carboplatin + oral topotecan; cisplatin + irinotecan	
	Carboplatin/cisplatin + etoposide + toripalimab followed by maintenance toripalimab	Platinum combination therapy: Cisplatin + irinotecan^a^; cisplatin + etoposide			
	Carboplatin/cisplatin + etoposide + tislelizumab followed by maintenance tislelizumab	**ECOG PS 0–2, >70 years of age:** Platinum combination therapy:			
	Carboplatin/cisplatin + etoposide	Cisplatin + etoposide; Carboplatin + etoposide or split cisplatin + etoposide			
	Carboplatin/cisplatin + irinotecan				
	Lobaplatin + etoposide				
	**ECOG PS 3–4 (non-SCLC related)**	**ECOG PS 3:**	**ECOG PS ≥2 (due to SCLC):**	**ECOG PS ≥2 (due to SCLC):**	
	BSC	Carboplatin + etoposide; split etoposide and cisplatin^c^	Carboplatin + etoposide	Carboplatin + etoposide; carboplatin + gemcitabine	
		**ECOG PS 4:**	**ECOG PS ≥2 (due to comorbidities):**	**ECOG PS ≥2 (due to comorbidities):**	
		Drug therapy not recommended^c^	BSC	BSC	
2L and beyond (relapsed SCLC)					
	**Platinum-resistant (≤6 months relapse):**	**ECOG PS 0–1** Tarlatamab (3L or later)^c^ **ECOG PS 0–2, Platinum-resistant (<2–3 months relapse):**	**ECOG PS 0–2:**	**Platinum-resistant (<3 months TFI):** **ECOG PS 0–2:**	**ECOG PS 0–2:**
	Topotecan (oral or IV); clinical trial; irinotecan; paclitaxel; docetaxel; temozolomide; oral etoposide; vinorelbine; gemcitabine; lurbinectedin; bendamustine	Amrubicin monotherapy	Lurbinectedin^a^; topotecan (oral or IV)^a^; original 1L regimen excluding ICIs^a^; tarlatamab^a^; CAV^d^; oral etoposide^d^; paclitaxel^e^; docetaxel^e^; irinotecan^e^; temozolomide^e^; vinorelbine^e^; gemcitabine^e^; nivolumab^e^; bendamustine^e^	Topotecan (oral or IV); CAV; lurbinectedin	Tarlatamab^a^ (category 1^f^) clinical trial^a^; irinotecan^a^; lurbinectedin^a^; retreatment with platinum-based doublet; topotecan (oral or IV)^a^ Nivolumab or pembrolizumab (if not previously treated with an ICI); paclitaxel; temozolomide; CAV; docetaxel; gemcitabine; oral etoposide
	ECOG PS (0–2) (3L):		**ECOG PS >2:**	**ECOG PS >2 and/or refractory:**	
	Anlotinib		BSC	BSC; lurbinectedin	
	Clinical trial; nivolumab; pembrolizumab				
	**Platinum sensitive (>6 months relapse):**	**ECOG PS 0–2,** **Platinum sensitive (≥2–3 months relapse):**		**Platinum sensitive (≥3 months TFI):**	
	Original 1L regimen	Topotecan monotherapy; cisplatin + etoposide + irinotecan; amrubicin monotherapy; carboplatin + etoposide		Rechallenge with platinum + etoposide; topotecan (oral or IV); CAV	
	Lurbinectedin				

^a^Preferred regimen; ^b^If cisplatin is contraindicated; ^c^Weak recommendation; ^d^Taiwan FDA approved; ^e^Not approved by Taiwan FDA; ^f^High level evidence.

BSC, best supportive care; CAV, cyclophosphamide, doxorubicin, and vincristine; CTFI, chemotherapy-free interval; ECOG PS, Eastern Cooperative Oncology Group performance status; ES-SCLC, extensive-stage SCLC; FDA, US Food and Drug Administration; ICI, immune checkpoint inhibitor; IO, immunotherapy; IV, intravenous; L, line of therapy; LS-SCLC, limited-stage SCLC; SCLC, small cell lung cancer; TFI, treatment-free interval; US, United States.

#### LS-SCLC

Surgical resection of SCLC (stage I–II) as part of multimodality treatment remains controversial, with only a minority of patients being eligible (21). When permitted, guidelines across East Asia and Europe/North America suggest a similar approach, with extensive pathological mediastinal staging as a first step prior to surgery, followed by postoperative systemic therapy. Japanese guidelines strongly recommend surgical resection in stages I–IIA without lymph node metastases; for nonsurgical patients with an Eastern Cooperative Oncology Group performance status (ECOG PS) of 0–2, accelerated hyperfractionated irradiation is recommended, with stereotactic irradiation weakly recommended for inoperable stages I–IIA without lymph node metastases (64). In the US, Korea, and Thailand, concurrent chemoradiation or stereotactic ablative radiotherapy (SABR) followed by systemic therapy is recommended for LS-SCLC (stage I–II, T1–2, N0, M0) in selected patients whose SCLC is medically inoperable or for whom a decision was made against performing surgery (62). As per NCCN Guidelines, advanced technologies, such as volumetric modulated arc therapy, are appropriate to use for delivering adequate tumor doses (62). In China, stereotactic body radiation therapy (SBRT)/SABR followed by chemotherapy, or chemotherapy with concurrent/sequential radiotherapy, is recommended for patients in LS-SCLC (stages I–IIA) who are unwilling to undergo surgery, and in inoperable LS-SCLC (stages I–IIA) (66).

In LS-SCLC (stages I–II), postoperative therapy using chemotherapy or definitive chemoradiotherapy is recommended across East Asia and Europe/North America, although the timing and choice of drugs varies between the regional guidelines (**Figure 1**, **Table 2**). Although most of the East Asian and European/North American guidelines recommend an etoposide-based platinum combination therapy in LS-SCLC, the Japanese guidelines recommend an etoposide- or irinotecan-based cisplatin combination therapy (**Table 2**). Consolidation therapy with durvalumab has also shown significant overall survival and progression-free survival benefits for patients with LS-SCLC (72) and is now included in guidelines (62, 73).

#### ES-SCLC

In ES-SCLC, the first-line therapy recommended by all guidelines is systemic treatment with a combination of chemotherapy and immunotherapy (21, 62, 64–66). For patients with ES-SCLC and ECOG PS 0–1, all guidelines recommend carboplatin/cisplatin-etoposide in combination with atezolizumab or durvalumab as the preferred first-line therapy (**Table 2**). However, if immunotherapy is contraindicated or patients have a poor ECOG PS (≥2), the preferred treatment across most guidelines is chemotherapy. Although there are similarities, guidelines across East Asia and Europe/North America differ in recommendations based on ECOG PS (**Table 2**).

### Prophylactic cranial irradiation in LS- and ES-SCLC

Japanese guidelines strongly recommend prophylactic cranial irradiation (PCI) in patients who achieve complete remission after initial treatment of localized tumors in LS-SCLC (GRADE IB; i.e., strong positive recommendation with moderate confidence) (64). In China, PCI is recommended in LS-SCLC (T1–2, N0) for patients with operable disease, and in those with inoperable disease with complete/partial responses after SBTR/SABR or chemoradiotherapy (level III [weak] recommendation) (66). For patients beyond T1–2, N0, PCI is recommended (level II) for those with complete/partial responses (66). As per ESMO and NCCN Guidelines, the role of PCI is not well defined in patients with stage I–II disease; therefore, PCI is not recommended in patients with a poor ECOG PS, in those who are at risk of neurocognitive decline, in frail patients, or in those who are ≥70 years of age (21, 62). NCCN Guidelines indicate that PCI can be considered in ES-SCLC, with the caveats indicated above (62). In Europe, ESMO Guidelines indicate that, for ES-SCLC, PCI is the standard treatment for patients with stage IV disease (<75 years of age with ECOG PS 0–2) without progression after first-line chemotherapy (level II recommendation [i.e., generally recommended treatment]) (21). In China, although PCI has been recommended, this is not a preferred treatment option for patients with ES-SCLC and PS 0–2 or PS3–4 (66).

### Recommended 2L therapy and beyond in relapsed SCLC

Across treatment guidelines, recommendations following relapse include rechallenge with platinum-based chemotherapy as well as the use of topotecan, irinotecan, lurbinectedin, or tarlatamab (21, 62). In relapsed SCLC, there is a lack of consensus on how to define sensitivity to platinum-based therapies across the guidelines. The Japanese guidelines define platinum sensitivity/resistance using a disease progression cutoff of 60–90 days (approximately 2–3 months) after the end of first-line platinum-based chemotherapy (64). The NCCN Guidelines no longer use platinum sensitivity/resistance to distinguish 2L+ treatment choices whereas the ESMO guidelines still define it with a 3-month cutoff (21, 62). Chinese guidelines use a cutoff of >6 months after the end of first-line platinum-based chemotherapy to define platinum-sensitive relapse and ≤6 months to define platinum-resistant relapse (66). A study from Japan reassessed the cutoff values in the post–immune checkpoint inhibitor (ICI) era and found that a 75-day cutoff after the end of first-line treatment was the most suitable for the prognostic classification of relapsed SCLC compared with traditional cutoffs (39).

Guidelines also differ in their recommendations for optimal treatment in relapsed SCLC (**Table 2**); several therapies are approved for second-line use, as summarized in **Table 3**. Tarlatamab is now included in the NCCN Guidelines as the only category 1 recommended preferred option for 2L+ SCLC; topotecan is recommended in platinum-resistant and platinum-sensitive SCLC by ESMO and NCCN; in contrast, the Japanese guidelines recommend the use of topotecan only in platinum-sensitive SCLC (21, 62, 64). In China, platinum rechallenge is recommended in platinum-sensitive SCLC and topotecan is recommended in platinum-resistant SCLC; other recommendations include irinotecan, taxanes (paclitaxel and docetaxel), gemcitabine, oral etoposide, vinorelbine, temozolomide, bendamustine, and lurbinectedin (66). Taxanes are also potential treatment options recommended by ESMO and NCCN Guidelines (21, 62, 74). Other NCCN-recommended therapies in platinum-resistant and platinum-sensitive SCLC include irinotecan and ICIs (**Table 2**).

**Table 3:** Approved treatments in SCLC by region/country.

Drug^a^	MOA	Line of therapy	China NMPA approval (121)	Japan MHLW approval (122)	Korea MFDS approval (123)	Singapore HSA (124)/ Malaysia NPRA approval (125)	Taiwan FDA approval (126)	Thailand FDA approval (127)	US FDA approval (128)	EMA approval (129)
Adebrelimab	Anti-PD-L1 mAb	1L, ES, with CT	Approved	None	None	None	None	None	None	None
Atezolizumab	Anti-PD-1 mAb	1L, ES, with CT (carboplatin and etoposide)	Approved	Approved	Approved	Approved	Approved	Approved	Approved	Approved
Benmelstobart	Anti-PD-L1 mAb	1L, ES, with anlotinib and CT	Approved	None	None	None	None	None	None	None
Durvalumab	Anti-PD-L1 mAb	1L, ES, with CT	Approved	Approved	Approved	Approved	Approved	Approved	Approved	Approved
Lobaplatin	DNA cross-linker	1L	Approved	None	None	None	None	None	None	None
Serplulimab	Anti-PD-1 mAb	1L, ES, with CT	Approved	None	None	None	None	Approved	Orphan drug designation	None
Toripalimab	Anti-PD-1 mAb	1L, ES, with CT	Approved	None	None	None	None	None	None	None
Tislelizumab	Anti-PD-1 mAb	1L, ES, with CT	Approved	None	None	None	None	None	None	None
Amrubicin	Topoisomerase II inhibitor	2L (relapsed SCLC)	None	Approved	None	None	None	None	None	None
Irinotecan	Topoisomerase I inhibitor	2L monotherapy (liposomal injection)	None	Approved	Approved	None	None	Approved	Fast-track designation	None
Tarlatamab	Bispecific T-cell engager targeting DLL3	2L+^b^, 3L+^c^	None	Approved	Approved	Approved (Singapore); None (Malaysia)	Approved	Approved	Accelerated approval	None
Topotecan	DNA topoisomerase inhibitor	2L	Approved	Approved	Approved	None (Singapore); Approved (Malaysia)	Approved	Approved	Approved	Approved
Lurbinectedin	RNA transcription inhibitor	2L+	None	None	Approved	Approved (Singapore); None (Malaysia)	Accelerated approval	None	Approved	None
Pembrolizumab	Anti-PD-1 mAb	2L+	None	None	None	None	Approval withdrawn	None	Voluntary withdrawal of accelerated approval	None
		Advanced solid tumors, following progression and with no satisfactory alternatives	None	Approved	None	None	Approved	None	Accelerated approval	None
Anlotinib	Multi-targeted TKI	3L+	Approved	None	None	None	None	None	None	None
Nivolumab	Anti-PD-1 mAb	3L+	None	None	None	None	None	None	Approval withdrawn	None

^a^These approvals are based on each country’s regulatory authority reports at the time of this manuscript’s development, and the stated line of therapy is an approximation if not explicitly stated in the regulatory label; please refer to official product labels for most current approval status and nuanced description of the approved indications by market; ^b^Singapore, Taiwan, Thailand and US; ^c^South Korea, Japan.

CT, chemotherapy; DLL3, delta-like ligand 3; EMA, European Medicines Agency; ES, extensive stage; FDA, US Food and Drug Administration; HSA, Health Sciences Authority; L, line of therapy; mAb, monoclonal antibody; MFDS, Ministry of Food and Drug Safety; MHLW, Ministry of Health, Labour and Welfare of Japan; MOA, mechanism of action; NMPA, National Medical Products Administration; NPRA, National Pharmaceutical Regulatory Agency; PD-1, programmed cell death protein 1; PD-L1, programmed cell death ligand 1; SCLC, small cell lung cancer; TKI, tyrosine kinase inhibitor; US, United States.

China is currently the only country to have a third-line therapy, anlotinib, approved for the treatment of SCLC (66). Although amrubicin monotherapy is recommended in platinum-resistant SCLC in Japan, other guidelines do not recommend its use (**Table 2**). Globally, despite guidelines recommending optimal treatment options, as well as approvals of new treatment options such as immunotherapy, disparities in cancer treatment availability, accessibility, and affordability among Asian countries have set a major drawback in tackling disease burden in this region (75).

### Real-world treatment patterns in East Asia versus Europe/North America

Data from real-world studies provide a glimpse into the variability of treatment usage in East Asia versus Europe/North America (**Table 4**). Prior to the approval of immunotherapy in SCLC, platinum-etoposide therapy was the most frequent first-line treatment across East Asia and Europe/North America (25, 30, 35, 41, 44, 76–80). In Korea and Thailand, platinum-etoposide use ranged from 61.3 to 81.4% between 2007 and 2016 in patients with LS- and ES-SCLC (41, 44, 77). Between 2014 and 2016, the use of platinum-etoposide was significantly more common in the US (87.0%) than in Europe (82.1%) and Japan (73.3%, P<0.05) in patients with ES-SCLC (76). Carboplatin-etoposide was the most common first-line regimen in Japan, Europe, and the US from 2014 to 2016, though the highest usage was in the US (60.4% vs 41.3% in Europe and 49.6% in Japan) (76). In comparison, cisplatin-etoposide was more frequently used in Europe (40.8%) than in the US (26.6%) or Japan (23.7%) (P<0.05) from 2014 to 2016 (76). In Korea and Taiwan, the cisplatin-etoposide combination was the most commonly used first-line platinum therapy from 2011 to 2016 (41, 81).

**Table 4:** Overview of real-world treatment patterns in SCLC^a^: East Asia versus Europe/North America.

Treatment	Proportion of patients receiving treatment			
	East Asia		Europe/North America
1L			
Etoposide only	Taiwan:	7.5% (81)	–
Platinum doublet	Thailand:	79.6% (LS-SCLC + ES-SCLC) (44)	–
Platinum + etoposide	China:	49.7% (35)	82.1–87.0% (76)
	Japan:	73.3–82.0% (76, 99)	65% (30)
	Korea:	81.4% (77)	
		61.3% (41)	
Carboplatin + etoposide	Japan:	49.6% (76)	41.3–61.8% (25, 76, 78, 79)
	Taiwan:	12.1% (LS-SCLC) (81)	
		10.4% (ES-SCLC) (81)	
Cisplatin + etoposide	Japan:	23.7% (76)	>10.0–43.0% (25, 76, 78, 79)
	Korea:	39.8% (41)	
	Taiwan:	78.6% (LS-SCLC) (81)	
		81.1% (ES-SCLC) (81)	
Platinum + irinotecan	Japan:	18–22.7% (76, 99)	0.5–2.0% (76)
	Korea:	14.8% (77)	
Platinum + etoposide + atezolizumab	China:	7.0–59.1% (35, 83)	–
Platinum + etoposide + durvalumab	China:	8.9% (35)	–
2L			
Amrubicin	Japan:	55.0% (46)	–
Anlotinib	China:	4.5% (35)	–
Carboplatin + etoposide	Taiwan:	2.0% (81)	>15.0–52.7% (78, 79)
CAV	–		3.0–24.9% (30, 78)
Etoposide based	Taiwan:	12.5% (81)	
Irinotecan	Japan:	7.2% (46)	3.0% (30)
Platinum doublet	–		8.0% (30)
Platinum + etoposide	China:	7.0% (35)	11.0–12.5% (76)
	Japan:	23.0% (76)	
	Korea:	6.0% (77)	
Platinum + irinotecan	China:	7.6% (35)	1.5–10.5% (76)
	Japan:	11.5% (76)	
	Korea:	15.3% (77)	
Topotecan	Japan:	3.5–5.2% (46, 76)	4.0–>20.0% (30, 78, 79)
	Taiwan:	63.6% (81)	
3L			
Anlotinib	China:	3.8% (35)	–
CAV	–		0.3–38.5% (30, 78, 79)
Irinotecan	Japan:	8.4% (46)	1.0–<5.0% (30, 79)
	Korea:	5.6% (77)	
Topotecan	Korea:	5.7% (77)	~20.0–38.5% (78, 79)

a All studies except for the two noted in the table are in ES-SCLC.

CAV, cyclophosphamide, doxorubicin, and vincristine; ES-SCLC, extensive-stage SCLC; L, line of therapy; LS-SCLC, limited-stage SCLC; SCLC, small cell lung cancer; US, United States.

Use of irinotecan in combination with platinum was a common first-line treatment in Japan (22.7%) but not in the US (2.0%) or in Europe (0.5%, P<0.0001) during 2014–2016 (76). The high usage of irinotecan in Japan is reflective of the Japanese treatment guideline recommendation (64). Irinotecan in combination with platinum is also used as a first-line and second-line chemotherapy in Korea (77). Amrubicin was the most commonly used second-line (55.0%) or later (22.0%) therapy compared with other chemotherapy regimens in Japan (46). Due to failure in achieving survival benefit in European/North American studies, amrubicin is currently not available in these regions (21, 61, 62).

Although limited data are available on the real-world usage of immunotherapy in SCLC owing to its approval only in the recent years, studies from Europe and China indicate an increased use of immunotherapy combination as first-line therapy in ES-SCLC in the post-approval era (82, 83). A pan-European study reported that, among patients with ES-SCLC receiving a first-line treatment (N = 1176), the use of platinum-based chemotherapy (platinum-etoposide) decreased from 91.8% in 2018 to 42.3% in 2021 (82). This decline was associated with an increased use of immunotherapy combination during the study period: the use of platinum-etoposide in combination with atezolizumab increased from 0% in 2018, reflecting the approval of atezolizumab only in late 2019 in Europe, to 41.2% in 2021 (82). A multicenter Chinese study in patients with ES-SCLC (N = 225) reported a higher proportion of patients receiving first-line platinum-etoposide in combination with atezolizumab (59.1%) versus platinum-etoposide alone (40.9%) during 2019–2022 (83).

## Prognosis and clinical outcomes in East Asia versus Europe/North America

### Differentiating key prognostic factors in SCLC: East Asia versus Europe/North America

Given SCLC has been viewed as a smoker’s disease, studies have looked at whether smoking could be a potential prognostic factor for poor survival outcomes. Studies from East Asia and Europe/North America report conflicting results for survival outcomes based on smoking history (28, 32, 41, 50). Most of the studies noted that there was no significant correlation between smoking status and overall survival (OS) in SCLC (LS- and ES-SCLC) (28, 32, 41). However, of note, the study by Liu et al. from China reported that, in LS- and ES-SCLC, smoking is an independent prognostic factor for poor progression-free survival (PFS) but not for OS in SCLC: in never-smokers versus smokers, median PFS was 8.37 versus 7.10 months (hazard ratio [HR], 0.753; P = 0.047), and median OS was 19.73 versus 14.40 months (HR, 0.780; P = 0.236), respectively (32). In the US study by Ou et al., a positive history of smoking was noted as a significant prognostic factor for poor OS in ES-SCLC (HR, 1.31; P = 0.0125; vs never-smokers) (50).

Studies have reported differences in OS and toxicity between East Asian and European/North American populations (84, 85). Ethnicity is a prognostic factor in SCLC, with studies indicating that better survival outcomes in SCLC are seen in patients of Asian ethnicity than in those of White ethnicity (86, 87). In LS-SCLC (stage III), Asian patients have a reduced risk of death compared with White patients (HR, 0.83; 95% CI, 0.77–0.91; P<0.001) (86). In ES-SCLC, Asian ethnicity is a favorable prognostic factor (HR, 0.785; P = 0.0076) (50). Other differentiating prognostic factors of note are high neutrophil–lymphocyte ratio and platelet–lymphocyte ratio, which have been associated with poor prognosis for survival outcomes in an East Asian population but not in a White population (88). Inherent differences due to genetic polymorphisms could also affect drug metabolism, transport, and receptor-binding (85).

### Survival outcomes by region/ethnicity in clinical trials

Ethnicity-related differences were noted in response to chemotherapy in two large phase 3 trials from Japan (JCOG 9511) and North America (SWOG 0124), despite similar eligibility criteria and treatment regimens (89). The two studies compared the survival benefit of cisplatin-etoposide with that of cisplatin-irinotecan in ES-SCLC, with the Japanese study showing a survival benefit for cisplatin-irinotecan (90, 91). On the contrary, the North American trial, consisting of more than 90.0% White patients, did not report any difference in survival outcomes for cisplatin-irinotecan versus cisplatin-etoposide (91).

A pooled comparative outcomes analysis of these two studies noted significantly higher response rates in the Japanese study population compared with the North American study population: 68.0% versus 57.0% (P = 0.02) in the cisplatin-etoposide group, and 87.0% versus 60.0% (P<0.001) in the cisplatin-irinotecan group, respectively (89). OS and PFS were similar across the two studies in the cisplatin-etoposide group. However, OS was significantly higher in the cisplatin-irinotecan group in the Japanese patients versus North American patients (12.8 vs 9.9 months; P<0.001, adjusted for age, sex, and ECOG PS). Differences in toxicity were also noted across the two studies, with Japanese study patients experiencing increased hematologic toxicity versus US study patients (89). However, it is crucial to consider that, besides pharmacogenomic variability among various ethnicities, differences between clinical trials investigating similar or identical therapies could also be a result of many other factors, including differences in study design, eligibility criteria, patient selection, demographics, and treatment regimens (91).

Results from phase 3 trials that evaluated immunotherapy plus chemotherapy, such as CASPIAN and IMpower133, demonstrated similar efficacy outcomes in their global trials and corresponding Asian subgroup analyses (92–96). CASPIAN and IMpower133 studies evaluated the programmed cell death protein 1 (PD-1) inhibitors durvalumab and atezolizumab, respectively, in a first-line treatment setting for ES-SCLC. In the CASPIAN global trial, durvalumab plus platinum-etoposide significantly improved OS compared with platinum-etoposide alone (median, 12.9 vs 10.5 months; HR, 0.75; 95% CI, 0.62–0.91; nominal P = 0.0032) (92). Results from a preplanned subgroup analysis of Japanese patients, as well as an exploratory analysis of a subgroup of Asian patients (Japan, South Korea, Taiwan, or China), were similar to the global data (93, 94). In both of these studies, durvalumab plus platinum-etoposide numerically improved OS versus platinum-etoposide alone: median not reached versus 15.2 months (HR, 0.77; 95% CI, 0.26–2.26) in Japanese patients (93), and 14.8 versus 11.9 months (HR, 0.87; 95% CI, 0.45–1.64) in the Asia subgroup, respectively (94). Based on interim results from the ongoing phase 3 ADRIATIC trial, adjuvant therapy with durvalumab in patients with LS-SCLC was shown to significantly improve OS (median, 55.9 vs 33.4 months; HR, 0.73; 98.321% CI, 0.54–0.98; P = 0.01) and PFS (median, 16.6 vs 9.2 months; HR, 0.76; 97.195% CI, 0.59–0.98; P = 0.02) compared with placebo (72). Of note, nearly half of the study population in the ADRIATIC trial are Asian (72). In the global IMpower133 trial, atezolizumab in combination with carboplatin-etoposide significantly improved OS (median, 12.3 vs 10.3 months; HR, 0.70; 95% CI, 0.54–0.91; P = 0.007) and PFS (median 5.2 vs 4.3 months; HR, 0.77; 95% CI, 0.62–0.96; P = 0.02) compared with chemotherapy alone (95). Results from a subgroup analysis in Japanese patients were consistent with the global trial (96).

### Survival outcomes in real-world studies: East Asia versus Europe/North America

Real-world cancer registry data from Japan reported a 5-year survival rate of approximately 20.0% for patients with localized SCLC and 2.0% for those at an advanced stage of the disease (97). Five-year relative survival rates from US-based registry data also demonstrate a similar trend, ranging from 30.0% for those with localized SCLC to 3.0% for those with metastatic disease (98). It remains unclear if these outcomes are impacted by differences in the usage of treatments between regions due to a lack of treatment utility data.

OS data from real-world studies across East Asia and Europe/North America, stratified by line of therapy and treatment, are provided in **Table 5**. For patients with LS-SCLC, OS ranged from 9.3 to 22.2 months in East Asia; data for Europe/North America were limited (**Table 5**). The ranges for OS in patients with ES-SCLC were 4.2–15.8 months in East Asia and 2.9–12.8 months in Europe/North America.

**Table 5:** OS outcomes from real-world studies in SCLC: East Asia versus Europe/North America^a^.

Treatment	East Asia		Europe/North America
1L			
Cisplatin + etoposide + durvalumab	China:	14.8 (ES-SCLC) (130)	–
Etoposide only	Taiwan:	4.2 (ES-SCLC) (81)	–
		9.3 (LS-SCLC) (81)	
Platinum + etoposide	**Range:**	7.2–13.6 (ES-SCLC)	**Range:** 7.0–12.5 (ES-SCLC)
Carboplatin/cisplatin based	Korea:	9.5 (ES-SCLC) (77)	7.1 (ES-SCLC) (31)
		8.5 (ES-SCLC) (40)	8.4 (ES-SCLC) (30)
	Japan:	13.6 (ES-SCLC, amrubicin 2L therapy) (99)	
Carboplatin + etoposide	Taiwan:	15.6 (LS-SCLC) (81)	19.2 (LS-SCLC) (100)
		7.2 (ES-SCLC) (81)	7.0 (mostly ES-SCLC) (80)
			7.9 (ES-SCLC) (78)
			8.0 (ES-SCLC) (26)
			9.3 (ES-SCLC) (25)
Cisplatin + etoposide	Taiwan:	16.8 (LS-SCLC) (81)	22.3 (LS-SCLC) (100)
		8.4 (ES-SCLC) (81)	8.22 (ES-SCLC) (78)
			9.0 (ES-SCLC) (26)
			9.6 (mostly ES-SCLC) (80)
			12.5 (ES-SCLC) (25)
Platinum + irinotecan	Korea, Japan:	10.8 (ES-SCLC) (46, 77)	–
Platinum + etoposide + atezolizumab	Korea:	12.0 (ES-SCLC) (131)	12.8 (ES-SCLC) (31)
		15.2 (ES-SCLC) (40)	
	Japan:	15.8 (ES-SCLC; trial-eligible population)	
		13.1 (ES-SCLC; trial-ineligible population) (37)	
2L			
Amrubicin	Japan:	10.0 (ES-SCLC) (46)	–
		14.0 (ES-SCLC) (99)	
CAV	–		3.34 (ES-SCLC) (78)
Platinum + etoposide	Korea:	22.2 (LS-SCLC) (20)	
		6.9 (ES-SCLC) (77)	
Carboplatin + etoposide			7.5 (ES-SCLC) (78)
Platinum + irinotecan	Korea:	6.6 (ES-SCLC) (77)	–
		16.4 (LS-SCLC) (20)	
Topotecan based	Korea:	5.1 (LS- and ES-SCLC) (81)	2.86 (ES-SCLC) (78)
3L			
CAV	–		2.89 (ES-SCLC) (78)
Topotecan	–		3.83 (ES-SCLC) (78)

a OS data presented as months.

CAV, cyclophosphamide, doxorubicin, and vincristine; ES-SCLC, extensive-stage SCLC; L, line of therapy; LS-SCLC, limited-stage SCLC; OS, overall survival; SCLC, small cell lung cancer.

The median OS of patients with ES-SCLC who received a platinum-etoposide first-line therapy ranged from 7.2 to 13.6 months in East Asia, and from 7.0 to 12.5 months in Europe/North America (25, 26, 30, 31, 40, 77, 78, 80, 81, 99). Some of the studies from East Asia and Europe/North America indicated a favorable survival outcome with first-line cisplatin-etoposide therapy in ES-SCLC (25, 81). Based on a multicenter Spanish observational study in ES-SCLC, cisplatin-etoposide therapy (first line) significantly increased median OS compared with carboplatin-etoposide therapy (12.5 vs 9.3 months, P<0.001) (25). Similarly, a Taiwanese study noted significantly improved OS in patients with ES-SCLC receiving first-line cisplatin-etoposide versus in those receiving carboplatin-etoposide (8.4 vs 7.2 months; P = 0.002) (81). A real-world Korean study in LS-SCLC showed that cisplatin-etoposide second-line therapy significantly improved OS compared with irinotecan-platinum therapy (22.2 vs 16.4 months, P<0.0001) (20). In the US, a real-world study in LS-SCLC demonstrated that OS was significantly improved in patients receiving first-line cisplatin-etoposide versus carboplatin-etoposide (22.3 vs 19.2 months, P = 0.017) (100).

Real-world data on immunotherapy in ES-SCLC remain scarce (40). On the basis of limited data available from East Asia and Europe/North America, there is a favorable survival trend for first-line immunotherapy in combination with chemotherapy in ES-SCLC when compared with chemotherapy alone (31, 40). In a Canadian study (N = 67), platinum-etoposide plus atezolizumab (first line) significantly prolonged OS compared with platinum-etoposide alone (12.8 vs 7.1 months; P = 0.01) (31). Similarly, a Korean study (N = 89) reported that first-line platinum-etoposide plus atezolizumab significantly improved OS compared with platinum-etoposide alone (15.2 vs 8.5 months; P = 0.047) (40).

## Implications and future perspectives

### Implications

Harmonization of SCLC guideline practices across Asian countries would require unified pan-Asian guideline recommendations for SCLC diagnosis and treatment that purposefully take into account the impact of ethnic, geographical, and socioeconomic factors (including differences in reimbursement policies) on clinical outcomes. Considering the complexity and diversity of these factors within Asia, the substantial regional collaboration and standardization efforts needed to create such guidance would present many challenges.

Ethnicity and smoking status are key patient characteristics that could potentially impact SCLC clinical outcomes across regions (12, 86). However, despite the differences in clinical outcomes observed between East Asia and Europe/North America, the outcomes themselves remain dismal across all regions. Although some studies indicate the potential impact of inter- and intra-population pharmacogenomic variability in treatment outcomes and/or toxicity, race- or ethnicity-based recommendations are under scrutiny, primarily because of “race” and “ethnicity” arguably being sociopolitical constructs rather than reflecting the true genotypic variations (89–91, 101, 102).

It is imperative, therefore, that researchers gain a better understanding of the impact genetic variations may have and what this may mean for the differential clinical management needed in SCLC. Many studies noted in this review do not report the ethnicity of the study population, and studies from some of the regions, particularly the US, have a mixed ethnicity-based population. Additionally, many regional studies were based on data from a single center or had small sample sizes, including subgroup analyses of clinical trials. Hence, it was challenging to draw any conclusive interpretations on the implications of ethnicity on SCLC clinical management. Moreover, data for East Asia might not be generalizable to the whole region, owing to scarce or no data from some of the East Asian countries, such as Singapore, Thailand, and Malaysia. Our review also highlights a lack of prevalence and incidence data specific to SCLC across various regions, as most registries only provide overall data on lung cancer, and data for SCLC are often reported on the basis of estimations. Moreover, most of the real-world evidence studies noted in this review were conducted prior to the approval of immunotherapy in SCLC. The impact of immunotherapy-based treatments on survival outcomes in patients with SCLC remains to be robustly evaluated.

### Emerging treatment options

A major challenge in the management of SCLC is the limited efficacy of existing treatments and the development of therapeutic resistance (103). Worldwide trends in SCLC survival analyses indicate that the prognosis of SCLC is still unsatisfactory, with no significant improvements in OS noted either in East Asia or in Europe/North America, thus highlighting the unmet need for the development of novel treatments (27, 97, 104, 105). Increased profiling of molecular subtypes in SCLC may be a promising avenue for the development of targeted therapies; however, further evidence is needed to support this personalized approach to treatment (57).

Delta-like ligand 3 (DLL3) has emerged as a promising candidate for targeted therapy in SCLC. As downregulation of major histocompatibility complex molecules, failure of antigen presentation, and tumor heterogeneity contribute to ICI resistance in SCLC, targeting alternative cell surface proteins provides a strategy for bypassing canonical antigen presentation pathways (1, 106, 107). DLL3-targeting therapeutic molecules currently in development include: T-cell engagers such as tarlatamab (half-life extended bispecific engager; phase 1–3 studies), Obrixtamig (BI764532; bispecific antibody; phase 1 study), and MK 6070 (HPN328; tri-specific recombinant protein; phase 1/2 study); and chimeric antigen receptor (CAR) therapies such as DLL3-CAR-NK cells (anti-DLL3–transduced natural killer cells; phase 1 study) (1). Of note, the US Food and Drug Administration recently granted tarlatamab accelerated approval for ES-SCLC with disease progression on or after platinum-based chemotherapy, following results of the DeLLphi-301 trial, which demonstrated objective response rates of 40% and OS of 14.3 months in the 10-mg dose group (108, 109). Based on a long-term follow-up of a median of 13.6 months, efficacy outcomes continued to be favorable in the 10-mg tarlatamab dose group (objective response rate, 40.4%; OS, 15.2 months) (110).

Other potential candidates for targeted therapy in SCLC are B7-H3, a member of the B7 ligand family, and seizure-related 6 homolog (SEZ6): both overexpressed in tumor cells with limited heterogeneity (111, 112). Examples of drugs currently in development include antibody–drug conjugates (ADCs) such as ifinatamab deruxtecan (anti-B7-H3 ADC; NCT04145622) (111, 113). Additionally, data from East Asian populations, mostly from China, are emerging, including the ETER701 study (NCT04234607) investigating the combination of benmelstobart (a PD-L1 inhibitor) and anlotinib plus etoposide/carboplatin and the ASTRUM-005 trial (NCT04063163), investigating serplulimab plus chemotherapy for first-line treatment in ES-SCLC (114–117).

## Summary

International guidelines for SCLC are rooted in similar approaches, but with some East Asian guidelines falling short of reflecting the inherent regional/population-based variability. It must be noted that most clinical trials in oncology are still largely conducted in Europe/North America, with the vast majority of patient populations being White (118). Given the low representation of non-White ethnic populations in clinical trials, there is a need to move away from the “one-size-fits-all” approach often used in clinical trial design, to conduct more studies that represent diverse populations. Potential differences in the molecular profiles of patients with SCLC in East Asia and Europe/North America also warrant further study on novel therapeutic approaches (60). A realistic approach to this end might be for clinical trial designs to include umbrella trials based on molecular subtypes in SCLC. Other key areas of exploration include differences in the underlying pathophysiology in never-smokers versus smokers with SCLC to help guide optimal clinical management.

Finally, given that clinical trials are conducted in highly selective populations in a controlled environment, reported outcomes might not be sufficiently representative of those seen in routine clinical practice (119). Therefore, it is imperative to also gather real-world outcomes data. Differences noted in the real-world treatment patterns and survival outcomes in SCLC in this review may not yet be substantial enough to advocate changes in SCLC clinical management in East Asia versus Europe/North America. Socioeconomic factors are certainly critical components that decide the course of clinical management in SCLC; however, further studies are needed to gauge and improve medicine availability and affordability disparities, as well as treatment accessibility issues, noted in many East Asian countries (75).

## Glossary

ADC: antibody–drug conjugate
AJCC: American Joint Committee on Cancer
ASCL1: Achaete-scute homolog 1
CAR: chimeric antigen receptor
CT: computed tomography
DLL3: delta-like ligand 3
ECOG PS: Eastern Cooperative Oncology Group performance status
ES: extensive-stage
ESMO: European Society for Medical Oncology
HR: hazard ratio
ICI: immune checkpoint inhibitor
IHC: immunohistochemistry
INSM1: insulinoma-associated protein 1
LS: limited-stage
NCAM/CD56: neural cell adhesion molecule
NCCN^®^: National Comprehensive Cancer Network^®^
NE: neuroendocrine
NK: natural killer
NSCLC: non–small cell lung cancer
OS: overall survival
PCI: prophylactic cranial irradiation
PD-1: programmed cell death protein 1
PD-L1: programmed cell death ligand 1
PFS: progression-free survival
RB1: retinoblastoma 1
SABR: stereotactic ablative radiotherapy
SBRT: stereotactic body radiation therapy
SCLC: small cell lung cancer
SEZ6: seizure-related 6 homolog
TMB: tumor mutational burden
TNM: Tumor, Node, Metastasis
TP53: tumor protein 53
US: United States
VA: Veterans Administration
WHO: World Health Organization.
